# TRIM47-mediated Ubiquitination of p53 Controls Proliferative Progression and Stress Adaptation in Glioblastoma

**DOI:** 10.7150/ijbs.131392

**Published:** 2026-04-08

**Authors:** Dakun Pei, Xiaosong Hu, Wen Peng, Hongbo Chang, Yi Du, Man Xu, Ping Liang, Hongjuan Cui

**Affiliations:** 1 State Key Laboratory of Resource Insects, Medical Research Institute, Southwest University, Chongqing, 400716, China.; 2 Jinfeng Laboratory, Chongqing, 400716, China.; 3 Department of Neurosurgery, Children's Hospital of Chongqing Medical University, National Clinical Research Center for Children and Adolescents' Health and Diseases, Ministry of Education Key Laboratory of Child Development and Disorders, Chongqing Key Laboratory of Child Neurodevelopment and Cognitive Disorders, Chongqing, China.

**Keywords:** glioblastoma, TRIM47, cell proliferation, p53, ubiquitination, cell cycle

## Abstract

Glioblastoma (GBM) is a highly aggressive malignancy characterized by dysregulated cell proliferation and impaired stress-response control. Here, we identify the E3 ubiquitin ligase TRIM47 as a regulator of p53 proteostasis and proliferative signaling in GBM. Integrated bioinformatic analyses and immunohistochemistry revealed that TRIM47 is upregulated in GBM and associated with unfavorable survival. Functional assays demonstrated that TRIM47 depletion suppressed GBM cell proliferation and clonogenic growth, induced G1-phase arrest, and markedly inhibited intracranial tumor growth *in vivo*. Mechanistically, TRIM47 interacted with p53 through its RING-containing region and promoted K48-linked ubiquitination predominantly at lysine 319, leading to proteasome-dependent degradation of p53. Loss of TRIM47 results in stabilization of p53 protein, activation of p21, accumulation of DNA damage, and attenuation of cell-cycle progression. In GBM models exposed to temozolomide-induced genotoxic stress, TRIM47 expression was reduced whereas p53 signaling and DNA damage markers were elevated. Moreover, inhibition of PDK1 kinase activity impaired TRIM47-mediated p53 ubiquitination and enhanced p53-dependent stress responses. Collectively, these findings establish TRIM47 as a critical regulator of p53 proteostasis and cell-cycle progression in GBM, thereby maintaining proliferative fitness under genotoxic stress.

## 1. Introduction

Glioblastoma (GBM) represents the most aggressive subtype of diffuse gliomas, characterized by high malignancy, therapeutic intractability, and extremely poor prognosis 1-3. Temozolomide (TMZ), a DNA-alkylating agent, induces DNA damage and apoptosis in tumor cells, and surgical resection combined with TMZ chemotherapy can modestly extend patient survival. However, a defining biological feature of GBM is its ability to sustain cell-cycle progression and proliferative capacity under therapeutic and intrinsic stresses, including genotoxic stress, hypoxia, and oxidative stress 4, 5, ultimately leading to tumor recurrence and treatment failure 6. Understanding how glioblastoma cells sustain proliferative capacity and cell-cycle progression under therapeutic stress remains a major challenge in GBM therapy 7, 8.

The tumor suppressor p53 is a central regulator of DNA damage responses and cell-cycle control, and it plays a critical role in regulating cellular responses to genotoxic stress, including DNA damage-induced cell-cycle arrest and checkpoint activation 9-11. In GBM, alterations of the p53 pathway are common and occur through TP53 mutations or dysregulation of upstream regulators, thereby promoting uncontrolled proliferation and resistance to therapy. Beyond genetic alterations, p53 activity can also be functionally attenuated through post-translational mechanisms, particularly ubiquitination-mediated proteasomal degradation mediated by E3 ubiquitin ligases such as MDM2 12-14. However, whether additional E3 ligases participate in the fine-tuning of p53 stability in GBM remains incompletely understood.

Protein ubiquitination, particularly E3 ligase-mediated protein degradation, plays a pivotal role in tumorigenesis and cell-fate determination 15, 16, as well as in regulating signal transduction, innate immunity, intracellular trafficking, and DNA damage responses 17-19. Tripartite motif (TRIM) family proteins exert diverse biological functions through their E3 ligase activity, including regulation of cell proliferation, differentiation, development, cell death, tumorigenesis, and innate immunity 20. All TRIM proteins share a conserved N-terminal RING-finger domain composed of cysteine and histidine residues arranged in a “cross-brace” structure that coordinates two zinc ions, which is essential for recruiting ubiquitin-charged E2 enzymes (E2∼Ub) 21, 22. Among them, TRIM47 has been reported to be associated with tumor progression in several cancer types, with proposed roles in the regulation of signaling pathways and therapeutic responses 23-26. However, the biological function of TRIM47 in GBM, particularly in the context of DNA damage responses and chemotherapeutic stress, remains largely unexplored. Accumulating evidence indicates that kinase-mediated phosphorylation can modulate the activity of E3 ubiquitin ligases 27-30, thereby linking intracellular signaling pathways with protein homeostasis. Phosphoinositide-dependent kinase 1 (PDK1), a central regulator of AGC kinases, has been implicated in tumor progression and therapeutic adaptation in multiple cancer contexts 31-34.

Although extensive studies have focused on mechanisms underlying fully established TMZ resistance, considerably less is known about the early adaptive phase in which GBM cells exhibit reduced TMZ sensitivity while retaining plasticity in stress-response pathways. This early stage may reflect dynamic remodeling of proliferation- and cell-cycle-associated regulatory circuits rather than irreversible resistance. In this study, we investigated the role of TRIM47 in GBM progression, with a particular focus on its regulation of p53 stability, cell-cycle control, and proliferative adaptation under genotoxic stress. By integrating bioinformatic analyses, molecular and biochemical approaches, and TMZ-nonresponsive GBM models representing an early reduced-sensitivity state, we aimed to delineate how TRIM47-associated ubiquitination contributes to GBM cell proliferation and stress adaptation.

## 2. Results

### 2.1 TRIM47 is upregulated in glioblastoma and correlates with poor prognosis

**Figure 1:**
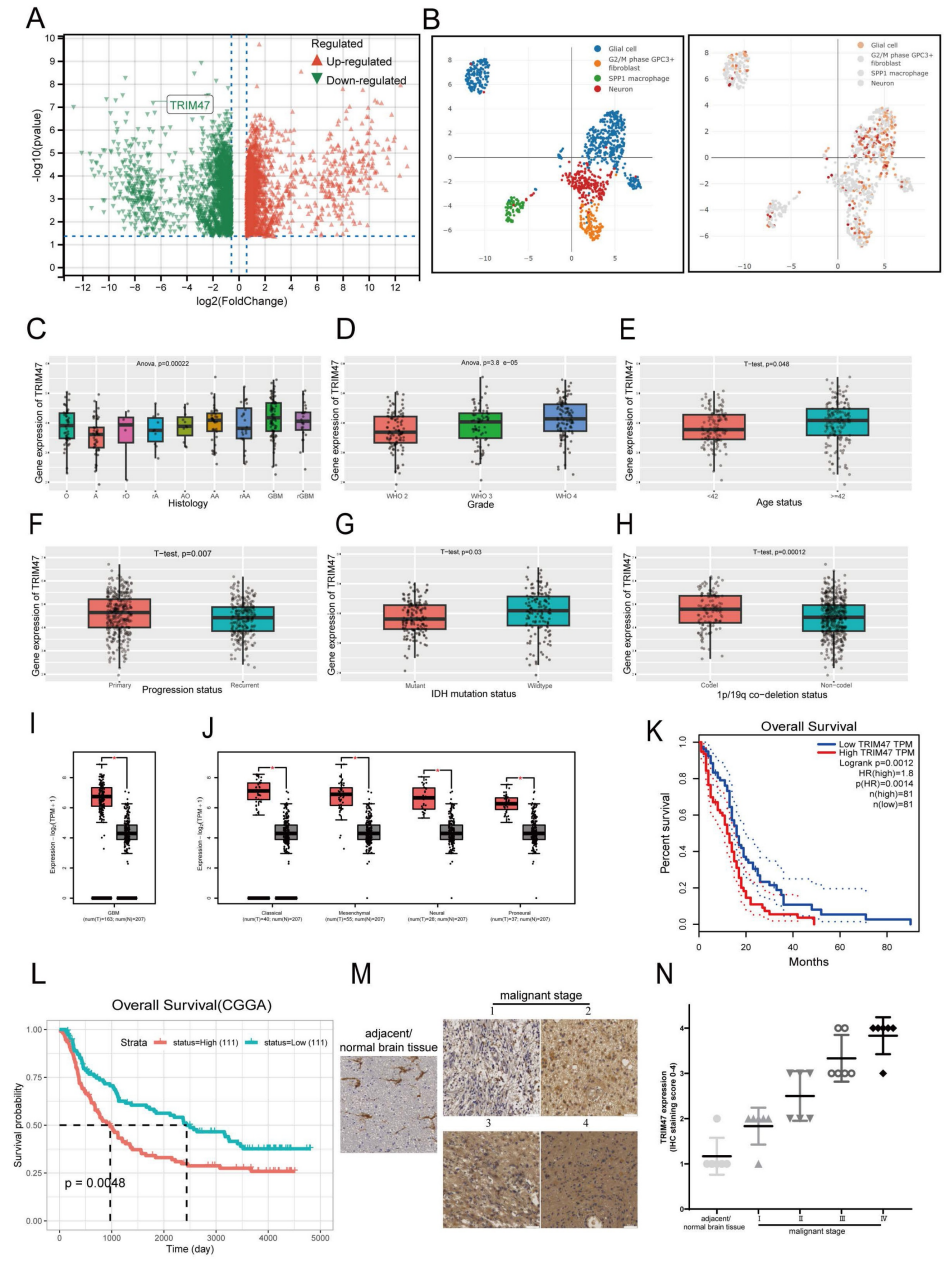
TRIM47 is highly expressed in glioblastoma and is associated with poor prognosis. (A) Quantitative proteomic analysis of U87MG and A172 cells during the acquisition of TMZ non-responsiveness, identifying TRIM47 as a differentially expressed protein. (B) Single-cell RNA-seq analysis from the DISCO database showing TRIM47 expression across annotated cell populations in a glioblastoma sample (sample GSM4483741; 1,546 cells). (C-H) TRIM47 expression stratified by clinicopathological variables (including primary vs recurrent status, WHO grade, age group, and IDH mutation status) was generated using the CGGA online portal. (I-J) TRIM47 expression across GBM molecular subtypes (classical, mesenchymal, neural, and proneural) was generated using the GEPIA portal based on TCGA-GBM data. Sample sizes for each subtype are indicated in the panels. (K) Kaplan-Meier overall survival (OS) analysis generated using GEPIA for TCGA-GBM patients stratified by TRIM47 expression (median cutoff, high vs low). P values were calculated by the log-rank test (time unit: months). (L) Kaplan-Meier survival analysis generated using the CGGA portal (dataset: mRNAseq_325; primary glioma; all WHO grades) stratified by TRIM47 expression, with group sizes indicated in the plot (high n = 111, low n = 111). P values were calculated by the log-rank test (time unit: days). (M-N) Representative immunohistochemical staining images and corresponding quantitative analysis of TRIM47 expression in normal brain tissues and glioma specimens of different WHO grades. Data are presented as mean ± SD where applicable. Survival P values were obtained by log-rank tests as indicated. P < 0.05 was considered statistically significant.

To explore molecular alterations associated with reduced sensitivity to genotoxic stress, U87MG and A172 cells were continuously exposed to gradually increasing concentrations of temozolomide (TMZ), followed by clonal selection to establish NR cell lines (NR, TMZ-nonresponsive), termed NR-A172 and NR-U87MG. Quantitative proteomic analysis indicated that TRIM47 expression was reduced during the acquisition of decreased TMZ responsiveness (Fig. 1A). These findings prompted us to further investigate the expression pattern and clinical relevance of TRIM47 in glioblastoma (GBM). To this end, we systematically analyzed the expression profile of TRIM47 across multiple publicly available datasets. Pan-cancer analysis using The Cancer Genome Atlas (TCGA) revealed that TRIM47 was significantly upregulated in GBM compared with other cancer types (Fig. S1A). Single-cell RNA-sequencing data from the DISCO database showed that TRIM47 expression was relatively enriched in glial cell populations, suggesting a cell-type-associated expression pattern within the tumor microenvironment (Fig. 1B, Fig. S1B). Analysis of the Chinese Glioma Genome Atlas (CGGA) dataset further showed that TRIM47 expression was significantly elevated in both primary and recurrent GBM samples. Notably, while TRIM47 expression remained elevated in recurrent tumors, a modest reduction compared with primary GBM samples was observed (Fig. 1C). In addition, TRIM47 expression was systematically evaluated across multiple clinicopathological parameters, including tumor grade, patient age, and IDH mutation status, revealing distinct expression patterns under different pathological conditions (Fig. 1D-H).

Integrated analysis of TCGA and Genotype-Tissue Expression (GTEx) datasets further confirmed the aberrantly high expression of TRIM47 in GBM. Moreover, TRIM47 was consistently highly expressed across all four molecular subtypes of GBM, including the classical, mesenchymal, neural, and proneural subtypes (Fig. 1I-J). We next evaluated the prognostic significance of TRIM47 in GBM. Kaplan-Meier survival analyses based on both the CGGA and TCGA cohorts revealed that elevated TRIM47 expression was significantly associated with poorer overall survival in GBM patients (Fig. 1K, L). To further validate these bioinformatic findings at the tissue level, immunohistochemical (IHC) staining was performed on tissue microarrays comprising normal brain tissues and glioma specimens of different grades. The IHC results showed that TRIM47 expression was higher in glioma tissues than in normal brain tissues and displayed an overall increasing trend across glioma grades (Fig. 1M, N).

Collectively, these data indicate that TRIM47 expression is dysregulated in GBM and is associated with tumor progression and patient prognosis.

### 2.2 Depletion of TRIM47 inhibits GBM cell proliferation

**Figure 2:**
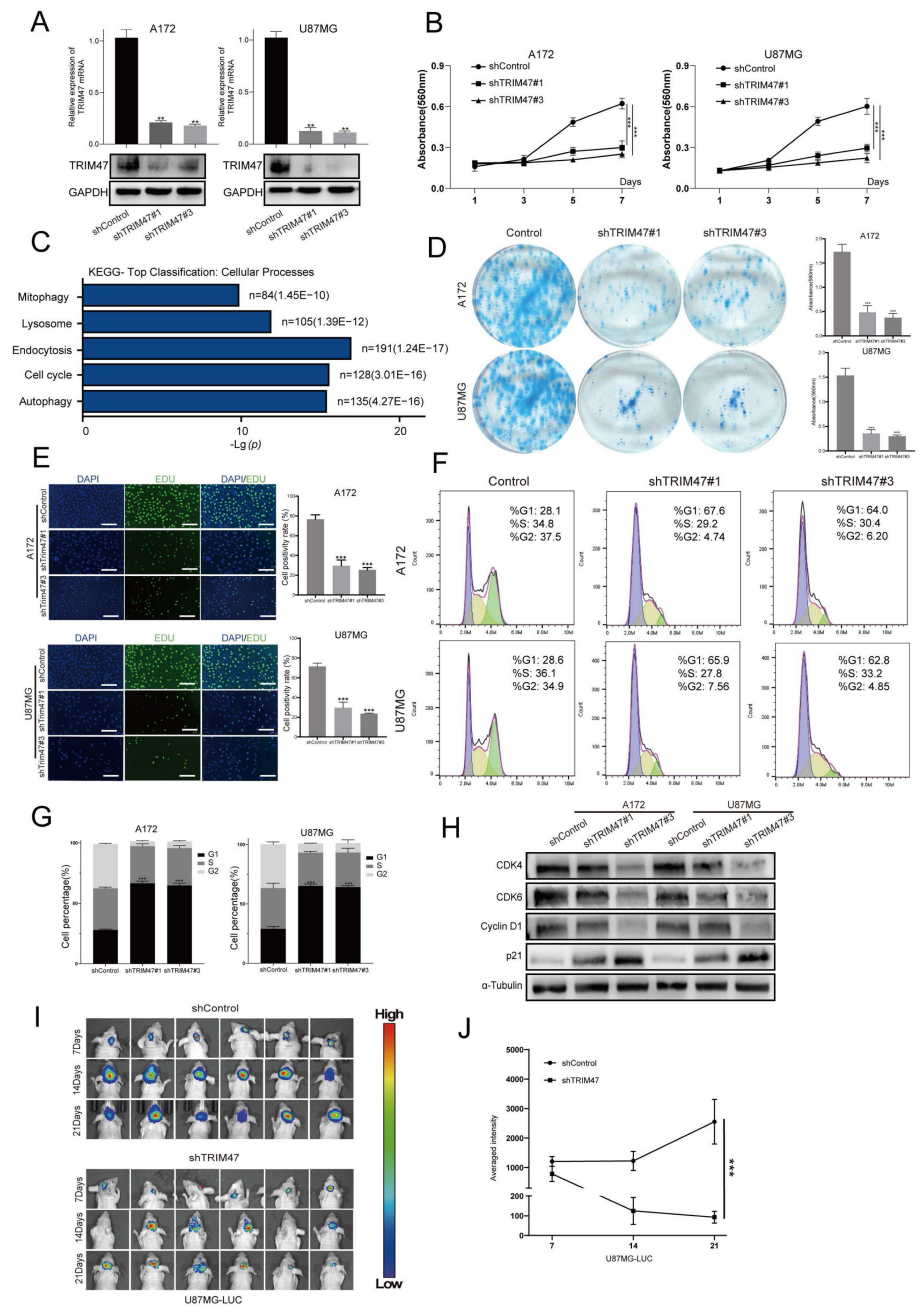
TRIM47 promotes glioblastoma cell proliferation and cell-cycle progression. (A) qRT-PCR and Western blot analyses confirming efficient TRIM47 knockdown in A172 and U87MG cells. (B) Cell Counting Kit-8 (CCK-8) assays showing reduced proliferative capacity following TRIM47 depletion. (C) KEGG pathway enrichment analysis of differentially expressed proteins in TRIM47-silenced cells. (D) Colony formation assays demonstrating impaired clonogenic capacity upon TRIM47 knockdown. (E) EdU incorporation assays indicating reduced DNA synthesis after TRIM47 depletion. (F-G) Flow cytometric analysis showing accumulation of cells in the G1 phase following TRIM47 knockdown. (H) Western blot analysis of cell-cycle-related proteins after TRIM47 depletion. (I-J) Bioluminescent imaging and quantitative analysis showing reduced intracranial tumor growth in mice implanted with TRIM47-depleted U87MG cells (N=6 mice per group). Data are presented as mean ± SD from at least three independent experiments. Student's t-test was used for statistical analysis. P < 0.05 was considered statistically significant.

To further elucidate the functional role of TRIM47 in GBM progression, two independent short hairpin RNAs targeting distinct regions of TRIM47 were employed to suppress TRIM47 expression in A172 and U87MG cells (Table S1). Efficient knockdown of TRIM47 was confirmed at both the mRNA and protein levels by qRT-PCR and Western blot analysis, respectively (Fig. 2A). Cell Counting Kit-8 (CCK-8) assays demonstrated that depletion of TRIM47 significantly suppressed the proliferative capacity of both A172 and U87MG cells compared with control cells (Fig. 2B), indicating that TRIM47 positively regulates GBM cell proliferation. Notably, both shRNAs produced highly comparable inhibitory effects on cell growth, supporting a consistent functional contribution of TRIM47 in sustaining proliferative signaling.

To further explore the molecular mechanisms underlying this effect, we performed quantitative proteomic profiling followed by KEGG pathway enrichment analysis in TRIM47-depleted cells. Differentially expressed proteins were significantly enriched in pathways related to cell-cycle regulation and proliferation-associated programs (Fig. 2C). Functional validation assays further corroborated these observations. Plate colony formation assays demonstrated that TRIM47 knockdown markedly impaired the clonogenic capacity of GBM cells (Fig. 2D). Consistently, EdU incorporation assays revealed a significant reduction in DNA synthesis following TRIM47 depletion (Fig. 2E). Furthermore, flow cytometric analysis of cell cycle distribution showed that silencing TRIM47 resulted in a pronounced accumulation of cells in the G1 phase accompanied by a concomitant decrease in the G2 phase population (Fig. 2F, G). At the molecular level, Western blot analysis revealed that knockdown of TRIM47 led to a significant downregulation of key G1/S cell cycle regulators, including CDK4, CDK6, and Cyclin D1, whereas the cyclin-dependent kinase inhibitor p21 was markedly upregulated. These results collectively indicate that TRIM47 depletion impairs G1/S cell-cycle progression in GBM cells. The tumor-promoting effects of TRIM47 were further validated *in vivo* using an orthotopic xenograft model. U87MG-LUC cells with stable TRIM47 knockdown were intracranially implanted into nude mice, and bioluminescent imaging revealed a significant reduction in tumor growth and tumor burden compared with control mice (Fig. 2I-J). To explore whether TRIM47 may also influence GBM cell motility, Transwell migration and invasion assays were performed. TRIM47 knockdown was associated with reduced migration and invasion *in vitro* (Supplementary Fig. S2A-B).

Importantly, re-expression of TRIM47 in TRIM47-depleted cells largely restored cell proliferation and migration abilities (Fig. S3), further supporting the specificity of TRIM47-mediated effects. Collectively, these results demonstrate that TRIM47 functions as an important regulator of proliferative and invasive phenotypes in GBM cells.

### 2.3 TRIM47 depletion elevates p53 protein stability and induces DNA damage

**Figure 3:**
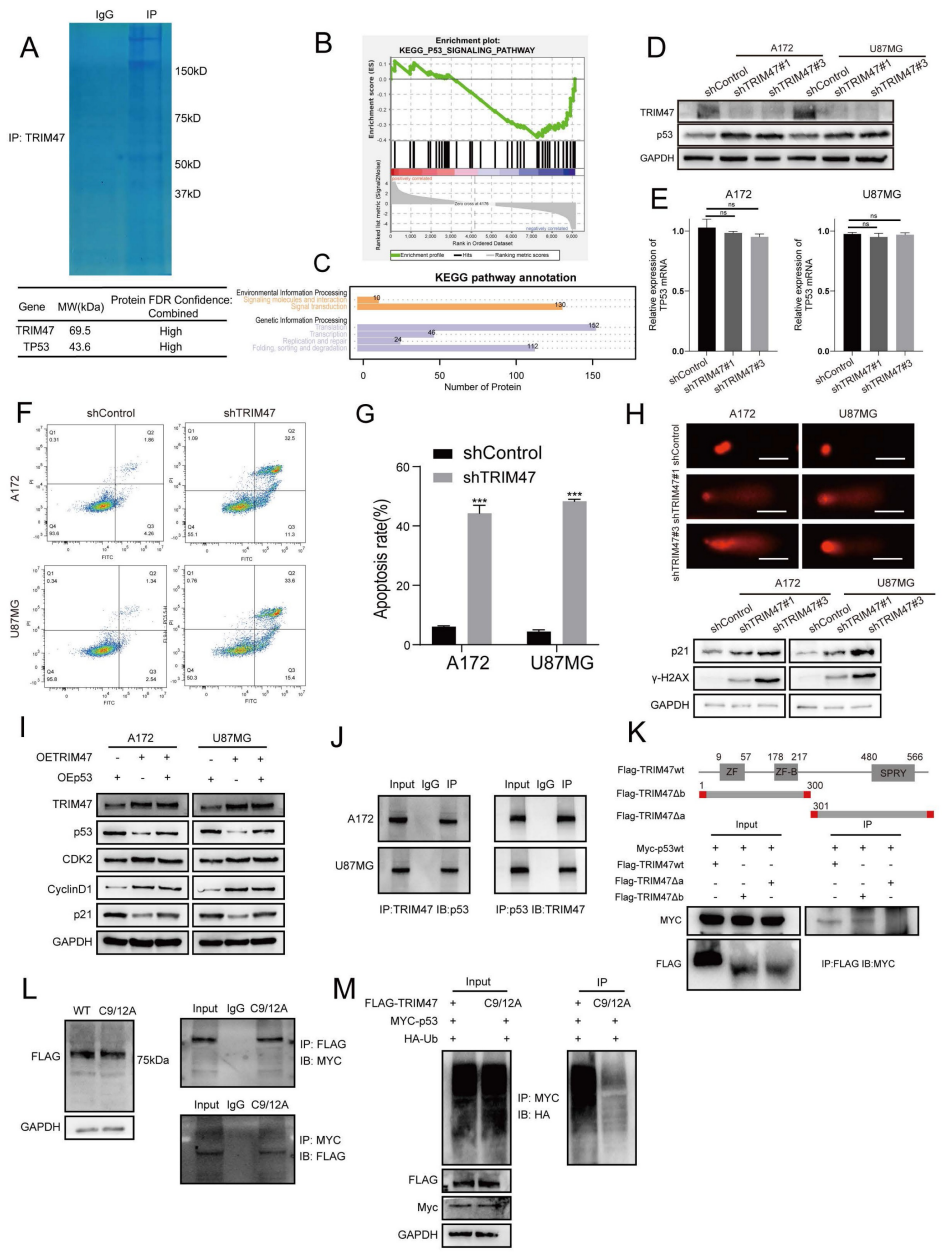
TRIM47 depletion stabilizes p53 and enhances DNA damage responses. (A) TRIM47-centered immunoprecipitation followed by mass spectrometry analysis identifying p53 as a potential interacting protein. (B) Gene set enrichment analysis (GSEA) of TMZ-related proteomic data showing significant enrichment of the p53 signaling pathway. (C) KEGG pathway enrichment analysis of TRIM47-associated proteins. (D-E) Western blot and qRT-PCR analyses of p53 protein and mRNA expression following TRIM47 knockdown. (F-G) Flow cytometric analysis demonstrating increased apoptotic cell populations in TRIM47-depleted GBM cells. (H) Comet assays showing enhanced DNA damage upon TRIM47 depletion. Western blot analysis of p21 and γ-H2AX expression following TRIM47 knockdown. (I) p53 restoration (epistasis) experiment under TRIM47 overexpression showing TRIM47, p53, p21, and cell-cycle-related proteins in A172 and U87MG cells. (J) Co-immunoprecipitation assays demonstrating an association between endogenous TRIM47 and p53. (K) Interaction analysis using TRIM47 truncation constructs indicating that p53 associates with a TRIM47 fragment containing the RING domain. (L) Expression of WT TRIM47 and the RING-dead mutant (C9A/C12A), and reciprocal co-immunoprecipitation assays assessing association with p53. (M) Ubiquitination assay comparing WT TRIM47 and the RING-dead mutant (C9A/C12A) in the presence of HA-ubiquitin.

To elucidate the molecular mechanism by which TRIM47 promotes GBM cell proliferation, we performed TRIM47-centered immunoprecipitation followed by mass spectrometry analysis, which identified p53 as a candidate interacting partner of TRIM47. KEGG pathway enrichment analysis of TRIM47-associated proteins revealed significant enrichment in pathways related to DNA damage repair and stress-response signaling (Fig. 3A, C). In parallel, gene set enrichment analysis (GSEA) based on the TMZ-treated proteomic dataset demonstrated significant enrichment of the p53 signaling pathway (Fig. 3B), suggesting a functional association between TRIM47 and p53 signaling during GBM progression and therapeutic stress adaptation. We therefore examined the effect of TRIM47 depletion on p53 expression and DNA damage accumulation. Western blot analysis showed that knockdown of TRIM47 resulted in a marked increase in p53 protein levels, whereas p53 mRNA expression remained largely unchanged, indicating that TRIM47 regulates p53 predominantly at the post-transcriptional level (Fig. 3D, E).

Consistent with enhanced p53 signaling, TRIM47 silencing led to a significant increase in apoptotic cell populations, as assessed by flow cytometry (Fig. 3F-G), and increased DNA damage, as evidenced by comet assays (Fig. 3H). At the molecular level, depletion of TRIM47 significantly upregulated the expression of p21, a canonical transcriptional target of p53, as well as γ-H2AX, a marker of DNA double-strand breaks (Fig. 3H), further supporting activation of a p53-associated DNA damage response upon TRIM47 knockdown. To test whether TRIM47-driven cell-cycle signaling is mediated through suppression of p53, we performed a p53 restoration experiment in A172 and U87MG cells. TRIM47 overexpression reduced p53 abundance and the canonical target p21, accompanied by increased levels of cell-cycle-related proteins, whereas co-expression of p53 in the TRIM47-overexpressing background restored p21 and partially reversed the TRIM47-associated cell-cycle protein changes (Fig. 3I). These data support that TRIM47 can regulate p53 pathway output and cell-cycle-related proteins at least in part through p53 suppression. We next examined the relationship between TRIM47 and p53. Co-immunoprecipitation assays were performed in GBM cells, confirming an association between endogenous TRIM47 and p53 (Fig. 3J). To map the interaction domain, a series of Flag-tagged TRIM47 truncation mutants were generated, including a fragment encompassing amino acids 1-300 containing the RING domain and a fragment spanning amino acids 301-638 containing the SPRY domain. Co-transfection of full-length or truncated TRIM47 constructs with Myc-tagged p53 in HEK293 cells revealed that a TRIM47 truncation fragment containing the RING domain retained the ability to associate with p53, suggesting that this region contributes to TRIM47-p53 complex formation (Fig. 3K). To directly test the requirement for RING catalytic activity, we generated a TRIM47 RING-dead mutant (C9A/C12A). Compared with WT TRIM47, the RING-dead mutant showed a markedly reduced capacity to promote p53 ubiquitination (Fig. 3L-M). Given that the RING domain constitutes the catalytic core of E3 ubiquitin ligases and that TRIM47 depletion did not affect p53 mRNA expression, these findings suggest that TRIM47 regulates p53 protein stability through a ubiquitination-dependent mechanism. Collectively, these data indicate that loss of TRIM47 stabilizes p53 protein, enhances DNA damage signaling, and promotes apoptotic responses in GBM cells.

### 2.4 TRIM47 regulates p53 protein stability by mediating K48-linked ubiquitination at lysine 319

**Figure 4:**
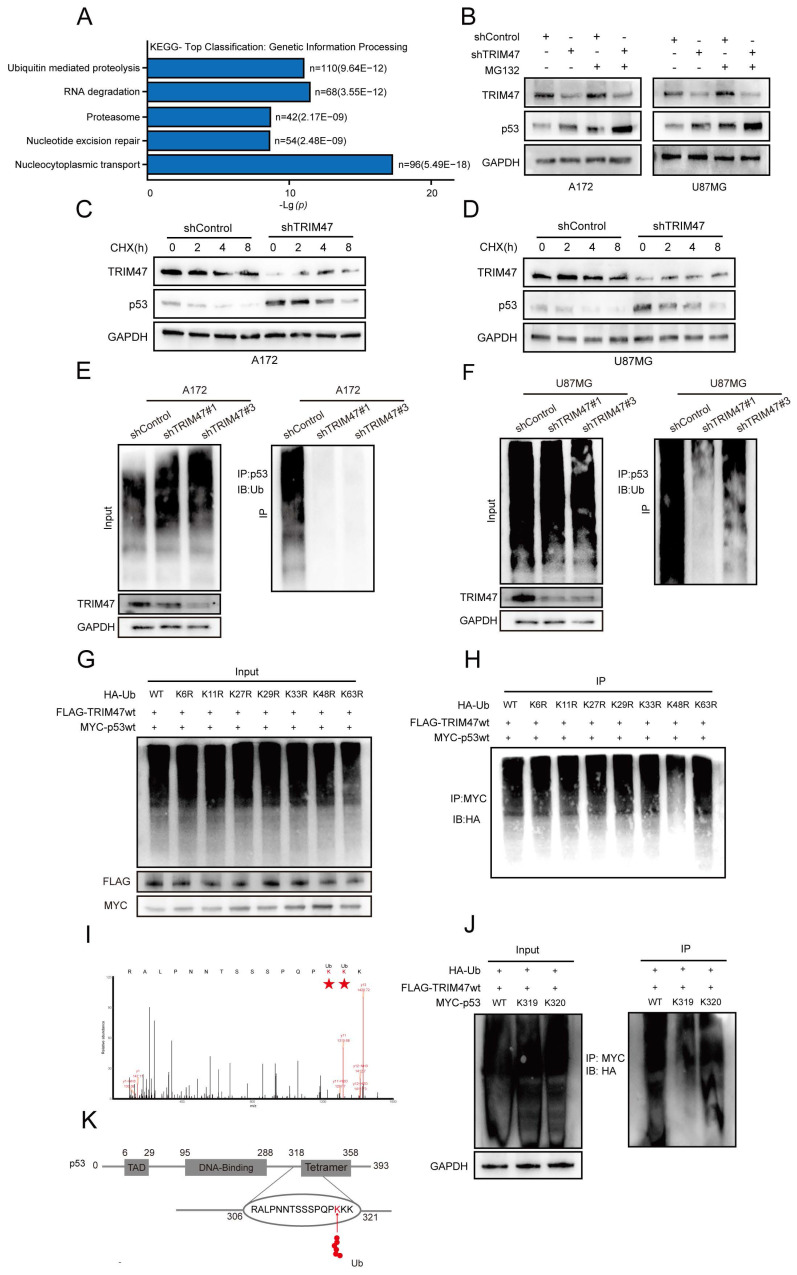
TRIM47 mediates K48-linked ubiquitination and proteasomal degradation of p53. (A) KEGG pathway enrichment analysis of proteomic data from TRIM47-depleted cells. (B) Western blot analysis showing accumulation of p53 following MG132 treatment in control and TRIM47-silenced cells. (C-D) Cycloheximide (CHX) chase assays assessing p53 protein stability following TRIM47 depletion. (E-F) Ubiquitination assays showing reduced p53 ubiquitination upon TRIM47 knockdown in A172 and U87MG cells. (G-H) Ubiquitin linkage-specific assays indicating preferential K48-linked ubiquitination of p53 mediated by TRIM47. (I-K) Identification and functional validation of p53 ubiquitination sites using mass spectrometry and site-directed mutagenesis.

Proteomic analysis of TRIM47-depleted GBM cells followed by KEGG pathway enrichment revealed significant enrichment of pathways related to ubiquitin-mediated proteolysis and DNA damage-associated processes, suggesting a potential role of TRIM47 in regulating protein stability under genotoxic stress (Fig. 4A). Given the observed association between TRIM47 and p53, we next examined whether TRIM47 regulates p53 protein turnover through the ubiquitin-proteasome system. Treatment of TRIM47-silenced A172 and U87MG cells with the proteasome inhibitor MG132 resulted in a pronounced accumulation of p53 protein. Notably, p53 levels were consistently higher in shTRIM47 cells than in control cells under MG132 treatment, indicating that loss of TRIM47 impairs proteasome-dependent degradation of p53 (Fig. 4B).

To directly assess the impact of TRIM47 on p53 protein stability, cycloheximide (CHX) chase assays were performed. These analyses showed that TRIM47 depletion significantly prolonged the half-life of p53 and delayed its degradation kinetics compared with control cells (Fig. 4C-D), supporting a role for TRIM47 in promoting p53 protein turnover. We next investigated whether the stabilization of p53 upon TRIM47 knockdown was attributable to altered ubiquitination. Ubiquitination assays revealed that depletion of TRIM47 markedly reduced the ubiquitination level of p53 in both A172 and U87MG cells (Fig. 4E-F), indicating that TRIM47 is required for efficient p53 ubiquitination in GBM cells. To determine the type of ubiquitin linkage involved, HEK293T cells were co-transfected with HA-tagged wild-type or lysine-mutant ubiquitin constructs (K6R, K11R, K27R, K29R, K33R, K48R, or K63R), together with Flag-tagged TRIM47 and Myc-tagged p53. TRIM47 robustly promoted ubiquitination of p53 in the presence of wild-type ubiquitin, whereas substitution of lysine 48, but not lysine 63, markedly attenuated this effect (Fig. 4G-H), indicating preferential involvement of K48-linked ubiquitination, which is classically associated with proteasome-dependent degradation. To further define the ubiquitination site(s) on p53 targeted by TRIM47, mass spectrometry analysis of p53 immunoprecipitates identified lysine residues K319 and K320 as candidate ubiquitination sites, as evidenced by di-glycine remnants. To functionally validate these sites, Myc-tagged p53 mutants harboring lysine-to-arginine substitutions at K319 or K320 were generated and subjected to ubiquitination assays. Mutation of p53 at K319 substantially impaired TRIM47-mediated ubiquitination, whereas mutation at K320 exerted a comparatively modest effect (Fig. 4I-K), identifying K319 as a major ubiquitination site contributing to TRIM47-mediated p53 regulation. Because p53 turnover is classically controlled by MDM2, we examined TRIM47 regulation of p53 under MDM2 inhibition. Notably, in the presence of Nutlin-3, TRIM47 overexpression still modulated p53 pathway markers and p53 ubiquitination (Supplementary Fig. S4).

Collectively, these data indicate that TRIM47 promotes K48-linked ubiquitination of p53, predominantly at lysine 319, thereby facilitating proteasome-dependent p53 degradation and attenuating p53-associated DNA damage signaling in GBM cells.

### 2.5 PDK1 activity modulates TRIM47-mediated p53 ubiquitination

**Figure 5:**
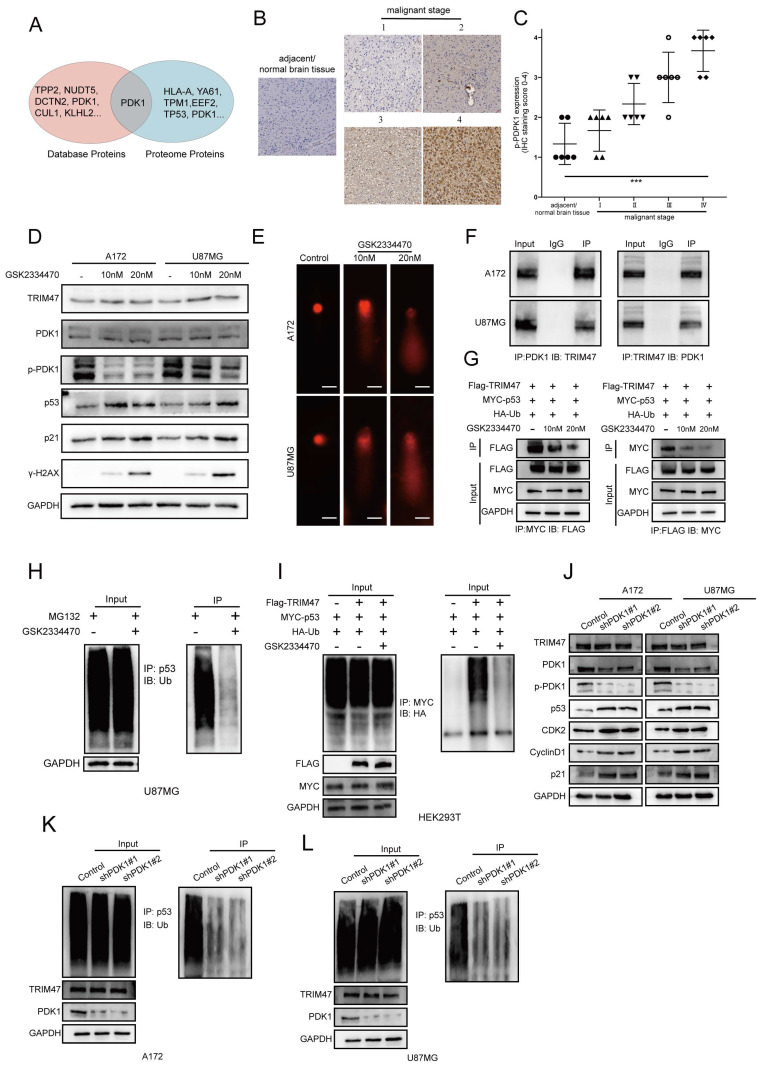
PDK1 activity modulates TRIM47-mediated p53 ubiquitination. (A) Protein interaction prediction and proteomic analyses suggesting a potential association between TRIM47 and PDK1. (B-C) Immunohistochemical staining images and quantitative analysis of PDK1 expression across glioma specimens of different WHO grades. (D) Western blot analysis showing increased p53, p21, and γ-H2AX expression following pharmacological inhibition of PDK1. (E) Comet assays demonstrating enhanced DNA damage upon PDK1 inhibition. (F) Co-immunoprecipitation assays showing an association between TRIM47 and PDK1. (G) Reduced association between TRIM47 and p53 following PDK1 inhibition. (H-I) Ubiquitination assays showing attenuation of TRIM47-mediated p53 ubiquitination after PDK1 inhibition. (J) Immunoblot analysis following genetic knockdown of PDK1 using two independent shRNAs in A172 and U87MG cells. (K-L) Ubiquitination assays showing reduced p53 ubiquitination following PDK1 knockdown in A172 and U87MG cells.

To explore potential upstream regulatory inputs that influence TRIM47-mediated p53 ubiquitination, we integrated database-based interaction prediction with proteomic and mass spectrometry analyses, which suggested an association between TRIM47 and 3-phosphoinositide-dependent protein kinase-1 (PDK1) (Fig. 5A). Immunohistochemical analysis of glioma tissue microarrays further showed that PDK1 expression positively correlated with tumor grade, supporting its involvement in GBM malignancy (Fig. 5B-C).

Given that kinase signaling frequently modulates the activity of E3 ubiquitin ligases, we examined whether PDK1 activity influences TRIM47-mediated regulation of p53. GBM cells were treated with GSK2334470, a selective inhibitor of PDK1 kinase activity. While TRIM47 protein levels remained largely unchanged, pharmacological inhibition of PDK1 activity resulted in a pronounced accumulation of p53, accompanied by increased expression of its downstream effector p21 and the DNA damage marker γ-H2AX (Fig. 5D). Consistently, comet assays demonstrated enhanced DNA damage following PDK1 inhibition (Fig. 5E). To assess whether PDK1 activity affects protein-protein interactions within the TRIM47-p53 module, co-immunoprecipitation assays were performed. Endogenous TRIM47 was found to associate with PDK1 in GBM cells (Fig. 5F). Notably, inhibition of PDK1 kinase activity weakened the interaction between TRIM47 and p53 without altering TRIM47 abundance (Fig. 5G), suggesting that PDK1 activity contributes to efficient complex formation between TRIM47 and p53. We next examined the functional consequence of PDK1 inhibition on p53 ubiquitination. Ubiquitination assays revealed that suppression of PDK1 activity significantly reduced TRIM47-dependent p53 ubiquitination in GBM cells (Fig. 5H). This effect was further confirmed in HEK293T cells co-transfected with HA-ubiquitin, Myc-p53, and Flag-TRIM47, in which GSK2334470 treatment markedly attenuated p53 ubiquitination (Fig. 5I). To complement pharmacological inhibition and mitigate potential off-target effects, we performed genetic knockdown of PDK1 using two independent shRNAs (Table S2). PDK1 depletion increased p53 and p21 levels and altered cell-cycle regulators (Fig. 5J). Consistently, PDK1 knockdown reduced p53 ubiquitination in both A172 and U87MG cells (Fig. 5K-L). These results provide genetic support that PDK1 activity contributes to regulation of the TRIM47-p53 ubiquitination axis in our system. Notably, PDK1 perturbation did not markedly change TRIM47 abundance but reduced TRIM47-p53 association and p53 ubiquitination, suggesting that PDK1 regulates this axis primarily by modulating interaction/ubiquitination efficiency rather than TRIM47 expression.

Together, these findings indicate that PDK1 kinase activity modulates the efficiency of TRIM47-mediated p53 ubiquitination, thereby influencing p53 stability and DNA damage signaling. Rather than acting as a direct determinant of TRIM47 expression, PDK1 appears to function as a regulatory input that fine-tunes TRIM47-p53 interactions in GBM cells.

### 2.6 PDK1 can still influence TRIM47-mediated ubiquitination of p53 under genotoxic stress from TMZ

**Figure 6:**
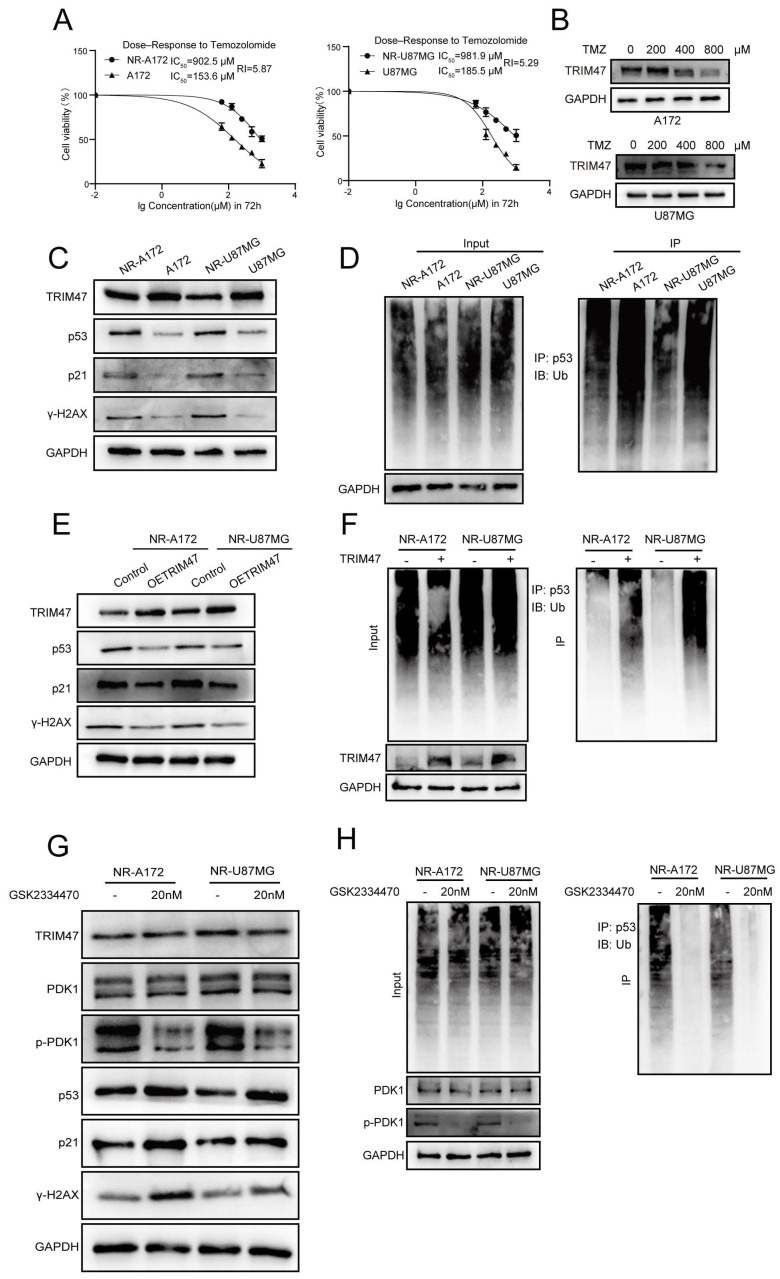
PDK1 regulates TRIM47-mediated ubiquitination of p53 and modulates cellular responses to temozolomide. (A) TMZ dose-response curves and IC₅₀ values in parental and TMZ-nonresponsive (NR) A172 and U87MG cells at 72 h. Dose-response curves were fitted by nonlinear regression (log[inhibitor] vs. response, variable slope) using GraphPad Prism. Data represent three independent experiments each performed with triplicate wells per concentration. (B) TRIM47 protein levels in TMZ-selected sublines generated during NR establishment under increasing maintenance TMZ concentrations (200, 400, and 800 μM) in A172 and U87MG cells. (C) Western blot analysis of TRIM47 and DNA damage-associated proteins in parental and NR cells. (D) Ubiquitination assays showing altered p53 ubiquitination in NR cells upon TRIM47 knockdown. (E-F) Restoration of TRIM47 expression suppresses p53 accumulation and DNA damage signaling in NR cells. (G-H) Effects of PDK1 inhibition on p53 stability, DNA damage markers, and p53 ubiquitination in NR cells.

To further investigate whether the PDK1-TRIM47-p53 axis is involved in modulating the cellular response to temozolomide, we established NR glioblastoma cell models by exposing A172 and U87MG cells to gradually increasing concentrations of TMZ followed by clonal selection. Compared with their parental counterparts, NR-A172 and NR-U87MG cells exhibited significantly elevated half-maximal inhibitory concentration (IC₅₀) values for TMZ, with resistance indices exceeding 5, indicating a reduced sensitivity to TMZ rather than fully irreversible resistance (Fig. 6A). Proteomic profiling indicated a reduction in TRIM47 expression during the acquisition of reduced TMZ sensitivity. We further examined staged TMZ-adapted sublines generated during NR establishment under increasing maintenance TMZ concentrations (200, 400, and 800 μM) and observed that TRIM47 began to decline from the 400 μM stage in both A172 and U87MG series (Fig. 6B). In parallel, the levels of p53 and its downstream effector p21 were substantially increased, accompanied by elevated γ-H2AX expression, suggesting enhanced DNA damage signaling in the NR state (Fig. 6C). Consistently, alkaline comet assays demonstrated significantly increased DNA damage in NR cells following TMZ exposure (Fig. S5A). These observations indicate that attenuation of TRIM47 expression is associated with p53 stabilization and activation of DNA damage responses during the acquisition of reduced TMZ sensitivity. To determine whether altered p53 stability in NR cells was linked to changes in TRIM47-mediated ubiquitination, ubiquitination assays were performed. Knockdown of TRIM47 in NR cells further reduced p53 ubiquitination levels, supporting a sustained dependence of p53 proteostasis on TRIM47 activity even in the NR context (Fig. 6D).

Conversely, ectopic re-expression of TRIM47 in NR-A172 and NR-U87MG cells led to a pronounced reduction in p53, p21, and γ-H2AX protein levels and significantly alleviated TMZ-induced DNA damage, as assessed by comet assays (Fig. 6E-F, Fig. S5B). These results suggest that restoration of TRIM47 expression partially counteracts DNA damage signaling in TMZ-challenged cells. Given the regulatory role of PDK1 in TRIM47-mediated p53 ubiquitination, we next examined whether pharmacological inhibition of PDK1 affects p53 stability and DNA damage responses in NR cells. Treatment with the selective PDK1 inhibitor GSK2334470 resulted in robust accumulation of p53 and γ-H2AX and a concomitant reduction in p53 ubiquitination in NR cells (Fig. 6G-H, Fig. S5C). Functionally, PDK1 inhibition significantly exacerbated DNA damage under TMZ treatment conditions, consistent with impaired TRIM47-dependent p53 degradation.

Collectively, these findings demonstrate that the PDK1-TRIM47-p53 axis dynamically regulates p53 stability and DNA damage responses during TMZ exposure. Rather than representing a static resistance mechanism, modulation of TRIM47 expression and activity appears to accompany an early reduced-sensitivity state during TMZ exposure that shapes TMZ sensitivity in glioblastoma cells. These findings warrant further investigation of PDK1 inhibition as a potential therapeutic strategy in GBM, especially in patients who have developed resistance to TMZ.

## 3. Discussion

Uncontrolled cell proliferation is a hallmark of glioblastoma and underlies its aggressive clinical behavior. In the present study, we identify TRIM47 as a regulator of proliferative capacity in GBM and demonstrate that TRIM47 promotes tumor growth by modulating p53 proteostasis. We show that TRIM47 is aberrantly upregulated in GBM, correlates with poor patient survival, and supports GBM cell proliferation, clonogenic growth and intracranial tumor expansion. These findings position TRIM47 as a malignancy-associated E3 ubiquitin ligase that contributes to the proliferative phenotype of GBM.

Mechanistically, our data indicate that TRIM47 directly associates with p53 through its RING-containing region and promotes K48-linked ubiquitination predominantly at lysine 319, leading to proteasome-dependent degradation of p53. Consistent with this mechanism, depletion of TRIM47 stabilized p53 protein without affecting TP53 mRNA expression, resulting in increased p21 expression, accumulation of DNA damage, and G1-phase cell-cycle arrest. These observations establish TRIM47 as a post-translational regulator of p53 that links ubiquitin-mediated proteolysis to cell-cycle progression in GBM cells. While MDM2 is a well-characterized E3 ligase for p53, our findings suggest that TRIM47 provides an additional layer of regulation that fine-tunes p53 stability and proliferative output in GBM. Clinical validation in larger, better-annotated cohorts is warranted to assess TRIM47-p53/p21 protein-level associations and multivariable survival analyses.

An important aspect of our study is the observation that TRIM47 expression is reduced in NR GBM models representing an early reduced-sensitivity state. This reduction is accompanied by p53 stabilization, activation of p21, and enhanced DNA damage signaling, suggesting that suppression of TRIM47-mediated p53 degradation may constitute part of an early reduced-sensitivity state under TMZ stress. Rather than reflecting fixed chemoresistance, this state appears to represent a dynamic phase in which proliferative control mechanisms are actively remodeled under genotoxic pressure. Restoration of TRIM47 expression partially reversed p53 accumulation and DNA damage signaling, further supporting a functional role for TRIM47 in regulating proliferative adaptation during TMZ exposure. MGMT is a major determinant of TMZ responsiveness, yet TMZ resistance is multifactorial and involves additional DNA repair and stress-adaptive programs. Because MGMT status can differ across GBM cells, we did not frame NR as a comprehensive model of all TMZ resistance mechanisms 35-37. Given that NR establishment under escalating TMZ pressure is likely multifactorial, we interpret the staged TRIM47 decrease as a dynamic change emerging during acquisition of reduced TMZ sensitivity rather than attributing it to a single mechanism. Instead, our data support that TRIM47 downregulation and p53 stabilization accompany an early reduced-sensitivity state, highlighting TRIM47-p53 proteostasis remodeling as an additional layer of stress adaptation. Validation across broader model systems with defined MGMT status will be required to further establish how this axis interacts with MGMT-dependent repair capacity in TMZ response.

We further demonstrate that TRIM47-mediated p53 ubiquitination is modulated by upstream kinase signaling. PDK1 is a central upstream kinase within the PI3K pathway that activates multiple AGC kinases and has been implicated in glioma growth and therapeutic adaptation. Prior studies have reported elevated PDK1 activity in gliomas and linked PDK1-dependent signaling to proliferative programs and treatment response. These observations provide a biological context for our findings 37-40. Pharmacological inhibition of PDK1 attenuated the interaction between TRIM47 and p53 and reduced TRIM47-dependent p53 ubiquitination, resulting in p53 stabilization and enhanced DNA damage responses. These findings indicate that TRIM47 does not function as a constitutively active oncogenic determinant but is instead subject to regulatory inputs that fine-tune its E3 ligase activity. Given the central role of PDK1 in coordinating proliferative signaling pathways, this regulatory axis may integrate growth signals with ubiquitin-dependent control of p53 and cell-cycle progression. Although our data support a functional requirement for PDK1 activity in promoting TRIM47-p53 complex formation and p53 ubiquitination, we did not directly test whether PDK1 phosphorylates TRIM47 or identify specific phosphorylation sites, which warrants future biochemical validation.

In summary, our study identifies TRIM47 as a regulator of p53-dependent cell-cycle control and proliferative capacity in GBM. By linking ubiquitin-mediated proteostasis to stress-responsive signaling, the PDK1-TRIM47-p53 axis contributes to proliferative adaptation under genotoxic stress. These findings expand current understanding of p53 regulation in GBM and suggest that targeting TRIM47-associated ubiquitination pathways may represent a potential strategy to modulate tumor cell proliferation in this highly aggressive malignancy.

## 4. Materials and Methods

### 4.1 Cell lines and cell culture

Human glioblastoma (GBM) cell lines A172 and U87MG, human embryonic kidney HEK293T cells, and the luciferase-labeled U87MG-LUC cell line were obtained from commercial sources. All cell lines were authenticated by short tandem repeat (STR) profiling and confirmed to be mycoplasma-free. Cells were cultured in high-glucose Dulbecco's Modified Eagle's Medium (DMEM; Gibco, USA) supplemented with 10% fetal bovine serum (FBS; Gibco, USA) at 37 °C in a humidified atmosphere containing 5% CO₂. A172 and U87MG are widely reported as TP53-wild-type glioblastoma cell lines 41-44.

### 4.2 Antibodies and reagents

Primary antibodies against TRIM47, PDK1, phospho-PDK1 (Ser241), p53, p21, CDK4, CDK6, Cyclin D1, γ-H2AX, E-cadherin, N-cadherin, Vimentin, MDM2 and MGMT were purchased from Proteintech (Wuhan, China). MG132, cycloheximide (CHX), temozolomide (TMZ), Nutlin-3, GSK2334470, and D-luciferin potassium salt were obtained from MedChemExpress (USA). Tissue microarrays were purchased from ZKGBio (Xi'an, China). All reagents were used according to the manufacturers' instructions.

### 4.3 Plasmids and lentiviral transduction

Short hairpin RNAs targeting TRIM47 and PDK1 (shTRIM47 and shPDK1), and control shRNA were cloned into the pLKO.1 vector. HA-tagged ubiquitin constructs (wild-type and lysine mutants K6R, K11R, K27R, K29R, K33R, K48R, and K63R), Flag-tagged TRIM47 (full-length and truncation mutants), and Myc-tagged p53 were generated using standard cloning strategies. Lentiviruses were packaged in HEK293T cells and used to infect GBM cells, followed by puromycin selection to establish stable cell lines.

### 4.4 Western blotting and immunoprecipitation

Cells or tissues were lysed in RIPA buffer supplemented with protease and phosphatase inhibitors. Proteins were separated by SDS-PAGE and transferred to PVDF membranes. After blocking, membranes were incubated with primary antibodies overnight at 4 °C and then with HRP-conjugated secondary antibodies. Signals were detected using enhanced chemiluminescence.

For immunoprecipitation assays, cell lysates were incubated with specific antibodies and protein A/G beads overnight at 4 °C. IgG controls and reciprocal immunoprecipitation assays were performed where indicated.

### 4.5 Ubiquitination and protein stability assays

For ubiquitination assays, HEK293T or GBM cells were co-transfected with the indicated plasmids and treated with MG132 for 7 h prior to harvest. Ubiquitinated proteins were detected by immunoprecipitation followed by immunoblotting. MG132 concentration was selected based on preliminary optimization to ensure effective proteasome inhibition without overt cytotoxicity. Protein stability was assessed using cycloheximide (CHX) chase assays, and protein levels were quantified by Western blotting.

### 4.6 Cell proliferation and cell cycle assays

Cell proliferation was assessed using CCK-8, colony formation, and EdU incorporation assays according to the manufacturers' instructions. Cell cycle distribution was analyzed by flow cytometry following propidium iodide staining. All experiments were performed in triplicate.

### 4.7 Quantitative reverse transcription PCR (qRT-PCR)

Total RNA was extracted using TRIzol reagent and reverse-transcribed into cDNA. qRT-PCR was performed using SYBR Green Master Mix, and relative gene expression was calculated using the 2⁻ΔΔCt method with GAPDH as the internal control.

### 4.8 Flow cytometry analysis

Apoptosis was evaluated using Annexin V-FITC/propidium iodide staining followed by flow cytometric analysis. Data were collected using a BD FACSCanto II system and analyzed with FlowJo software.

### 4.9 Establishment of TMZ-nonresponsive GBM cell lines

GBM cells were cultured in medium containing TMZ at their respective IC₅₀ concentrations. TMZ concentration was gradually increased over serial passages with monthly single-cell cloning and expansion. This process was continued until the resistance index (RI) exceeded 5, indicating the establishment of TMZ-nonresponsive cells exhibiting partial, reduced sensitivity rather than fully irreversible resistance.

### 4.10 Migration assays

Cell migration assays were performed using Transwell chambers with 8-μm pores. Migrated cells were fixed, stained, and quantified in randomly selected microscopic fields.

### 4.11 Comet assay

DNA damage was assessed using an alkaline comet assay according to the manufacturer's protocol. Tail moments were quantified using Comet Assay Software Project (CASP).

### 4.12 Orthotopic xenograft model and *in vivo* imaging

Female BALB/c nude mice were intracranially injected with U87MG-LUC cells to establish orthotopic GBM xenografts. Bioluminescence imaging was performed after intraperitoneal injection of D-luciferin using an IVIS Spectrum system. Imaging parameters were kept constant across experiments. All animal procedures were approved by the Institutional Animal Care and Use Committee.

### 4.13 Immunohistochemistry

Paraffin-embedded tissue sections were processed for immunohistochemical staining following standard protocols. Signals were visualized using DAB substrate and counterstained with hematoxylin.

### 4.14 Public database analysis

Public transcriptomic and clinical data were obtained from TCGA/GTEx via GEPIA and from CGGA (cohorts mRNAseq_325 and mRNAseq_693) via the CGGA online portal. In GEPIA, overall survival (OS) for TCGA-GBM was analyzed by Kaplan-Meier curves with median TRIM47 expression as the cutoff (high vs. low), with HRs/95% CIs estimated by the Cox PH model and P values calculated by the log-rank test (time unit: months). CGGA expression plots across clinicopathological strata were generated using the portal default settings; CGGA survival analysis shown in this study was generated in mRNAseq_325 (primary glioma, all WHO grades), with group sizes indicated in the plot (high n = 111, low n = 111; time unit: days). Single-cell RNA-seq analysis was performed using the DISCO database. TRIM47 expression across annotated cell populations was examined in a glioblastoma sample from GEO: GSE148842 (sample GSM4483741; brain tumor tissue; Drop-seq; vehicle-treated [DMSO]; 1,546 cells). Cell-type annotations provided by the database were used to compare TRIM47 expression between major cellular populations.

### 4.15 Statistical analysis

All experiments were performed with at least three independent biological replicates unless otherwise stated, and exact sample sizes (n) are provided in the corresponding figure legends. Data are presented as mean ± standard deviation (SD). For comparisons between two groups, an unpaired two-tailed Student's t-test was used. TMZ dose-response curves and IC₅₀ values were calculated by nonlinear regression (log[inhibitor] vs. response) in GraphPad Prism. Correlations were assessed using Pearson's or Spearman's correlation coefficients as appropriate. P < 0.05 was considered statistically significant, and significance levels are denoted as *P < 0.05, **P < 0.01, ***P < 0.001, and ****P < 0.0001. All statistical analyses were conducted using GraphPad Prism 9.0.

## Supplementary Material

Supplementary figures.

## Data Availability

All data supporting the findings of this study are available within the Article and Supplementary Information. Additional data-sharing information will be provided where applicable, in accordance with journal and public repository policies.

## References

[1] Ostrom QT, Price M, Neff C, Cioffi G, Waite KA, Kruchko C (2023). CBTRUS Statistical Report: Primary Brain and Other Central Nervous System Tumors Diagnosed in the United States in 2016-2020. Neuro Oncol.

[2] Louis DN, Perry A, Wesseling P, Brat DJ, Cree IA, Figarella-Branger D (2021). The 2021 WHO Classification of Tumors of the Central Nervous System: a summary. Neuro Oncol.

[3] Wei Y, Xu Y, Sun Q, Hong Y, Liang S, Jiang H (2024). Targeting ferroptosis opens new avenues in gliomas. Int J Biol Sci.

[4] Jin P, Feng X-D, Huang C-S, Li J, Wang H, Wang X-M (2024). Oxidative stress and cellular senescence: Roles in tumor progression and therapeutic opportunities. MedComm - Oncology.

[5] Ding X, Hou J, Hu X, Peng W, Li Y, Zhao G (2024). CoCl2-induced glioma hypoxia environment enhances CD47-SIRPα to promote tumor immune evasion. MedComm - Oncology.

[6] Ortiz R, Perazzoli G, Cabeza L, Jiménez-Luna C, Luque R, Prados J (2021). Temozolomide: An Updated Overview of Resistance Mechanisms, Nanotechnology Advances and Clinical Applications. Curr Neuropharmacol.

[7] Wang X, Lu J, Guo G, Yu J (2021). Immunotherapy for recurrent glioblastoma: practical insights and challenging prospects. Cell Death & Disease.

[8] Singh S, Dey D, Barik D, Mohapatra I, Kim S, Sharma M (2025). Glioblastoma at the crossroads: current understanding and future therapeutic horizons. Signal Transduction and Targeted Therapy.

[9] Guo Y, Wu H, Wiesmüller L, Chen M (2024). Canonical and non-canonical functions of p53 isoforms: potentiating the complexity of tumor development and therapy resistance. Cell Death & Disease.

[10] Chen Y, Ding K, Zheng S, Gao S, Xu X, Wu H (2025). Post-translational modifications in DNA damage repair: mechanisms underlying temozolomide resistance in glioblastoma. Oncogene.

[11] Lin T, Hou PF, Meng S, Chen F, Jiang T, Li ML (2019). Emerging Roles of p53 Related lncRNAs in Cancer Progression: A Systematic Review. Int J Biol Sci.

[12] Deng L, Meng T, Chen L, Wei W, Wang P (2020). The role of ubiquitination in tumorigenesis and targeted drug discovery. Signal Transduct Target Ther.

[13] Lee JT, Gu W (2010). The multiple levels of regulation by p53 ubiquitination. Cell Death Differ.

[14] Zhou Z, Qiu R, Liu W, Yang T, Li G, Huang W (2021). BCAS3 exhibits oncogenic properties by promoting CRL4A-mediated ubiquitination of p53 in breast cancer. Cell Prolif.

[15] Popovic D, Vucic D, Dikic I (2014). Ubiquitination in disease pathogenesis and treatment. Nat Med.

[16] Yang Y, Li C-CH, Weissman AM (2004). Regulating the p53 system through ubiquitination. Oncogene.

[17] Yang Q, Zhao J, Chen D, Wang Y (2021). E3 ubiquitin ligases: styles, structures and functions. Mol Biomed.

[18] Liu J, Cheng Y, Zheng M, Yuan B, Wang Z, Li X (2021). Targeting the ubiquitination/deubiquitination process to regulate immune checkpoint pathways. Signal Transduct Target Ther.

[19] Behera A, Reddy ABM (2023). WWP1 E3 ligase at the crossroads of health and disease. Cell Death & Disease.

[20] Chen R, Tie Y, Lu J, Li L, Zeng Z, Chen M (2022). Tripartite motif family proteins in inflammatory bowel disease: Mechanisms and potential for interventions. Cell Prolif.

[21] Cai C, Tang YD, Zhai J, Zheng C (2022). The RING finger protein family in health and disease. Signal Transduct Target Ther.

[22] Li Y, Wu H, Wu W, Zhuo W, Liu W, Zhang Y (2014). Structural insights into the TRIM family of ubiquitin E3 ligases. Cell Research.

[23] Liang Q, Tang C, Tang M, Zhang Q, Gao Y, Ge Z (2019). TRIM47 is up-regulated in colorectal cancer, promoting ubiquitination and degradation of SMAD4. J Exp Clin Cancer Res.

[24] Azuma K, Ikeda K, Suzuki T, Aogi K, Horie-Inoue K, Inoue S (2021). TRIM47 activates NF-κB signaling via PKC-ε/PKD3 stabilization and contributes to endocrine therapy resistance in breast cancer. Proc Natl Acad Sci U S A.

[25] Wang Y, Liu C, Xie Z, Lu H (2020). Knockdown of TRIM47 inhibits breast cancer tumorigenesis and progression through the inactivation of PI3K/Akt pathway. Chem Biol Interact.

[26] Li L, Yu Y, Zhang Z, Guo Y, Yin T, Wu H (2021). TRIM47 accelerates aerobic glycolysis and tumor progression through regulating ubiquitination of FBP1 in pancreatic cancer. Pharmacol Res.

[27] Yang Z, Yu Z, Teng J, Yanzhang R, Yu Y, Zhang H (2025). PDK1-mediated phosphorylation of USP5 modulates NF-κB signalling to enhance osteosarcoma growth. Int J Biol Macromol.

[28] Feng X, Zhang H, Meng L, Song H, Zhou Q, Qu C (2021). Hypoxia-induced acetylation of PAK1 enhances autophagy and promotes brain tumorigenesis via phosphorylating ATG5. Autophagy.

[29] Hepowit NL, Kolbe CC, Zelle SR, Latz E, MacGurn JA (2022). Regulation of ubiquitin and ubiquitin-like modifiers by phosphorylation. Febs j.

[30] Perez JM, Chen Y, Xiao TS, Abbott DW (2018). Phosphorylation of the E3 ubiquitin protein ligase ITCH diminishes binding to its cognate E2 ubiquitin ligase. J Biol Chem.

[31] Gagliardi PA, Puliafito A, Primo L (2018). PDK1: At the crossroad of cancer signaling pathways. Semin Cancer Biol.

[32] Liang Y, Chen P, Wang S, Cai L, Zhu F, Jiang Y (2024). SCF(FBXW5)-mediated degradation of AQP3 suppresses autophagic cell death through the PDPK1-AKT-MTOR axis in hepatocellular carcinoma cells. Autophagy.

[33] Zhou J, Yun E-J, Chen W, Ding Y, Wu K, Wang B (2017). Targeting 3-phosphoinositide-dependent protein kinase 1 associated with drug-resistant renal cell carcinoma using new oridonin analogs. Cell Death & Disease.

[34] Yang R, Wang M, Zhang G, Li Y, Wang L, Cui H (2021). POU2F2 regulates glycolytic reprogramming and glioblastoma progression via PDPK1-dependent activation of PI3K/AKT/mTOR pathway. Cell Death & Disease.

[35] Chahal M, Abdulkarim B, Xu Y, Guiot MC, Easaw JC, Stifani N (2012). O6-Methylguanine-DNA methyltransferase is a novel negative effector of invasion in glioblastoma multiforme. Mol Cancer Ther.

[36] Yi GZ, Huang G, Guo M, Zhang X, Wang H, Deng S (2019). Acquired temozolomide resistance in MGMT-deficient glioblastoma cells is associated with regulation of DNA repair by DHC2. Brain.

[37] Li H, Wu Y, Chen Y, Lv J, Qu C, Mei T (2025). Overcoming temozolomide resistance in glioma: recent advances and mechanistic insights. Acta Neuropathol Commun.

[38] Dewdney B, Jenkins MR, Best SA, Freytag S, Prasad K, Holst J (2023). From signalling pathways to targeted therapies: unravelling glioblastoma's secrets and harnessing two decades of progress. Signal Transduction and Targeted Therapy.

[39] Wu H, Gao W, Chen P, Wei Y, Zhao H, Wang F (2024). Research progress of drug resistance mechanism of temozolomide in the treatment of glioblastoma. Heliyon.

[40] Pournajaf S, Pourgholami MH (2025). The mTOR pathway in Gliomas: From molecular insights to targeted therapies. Biomedicine & Pharmacotherapy.

[41] Joruiz SM, Von Muhlinen N, Horikawa I, Gilbert MR, Harris CC (2024). Distinct functions of wild-type and R273H mutant Δ133p53α differentially regulate glioblastoma aggressiveness and therapy-induced senescence. Cell Death & Disease.

[42] Fiorenza I, Frederick ED, Elizabeth AK, Stacia LP, Michael AM (2007). Human Glioblastoma U87MG Cells Transduced with a Dominant Negative p53 (TP53) Adenovirus Construct Undergo Radiation-Induced Mitotic Catastrophe. Radiation Research.

[43] Sasaki A, Udaka Y, Tsunoda Y, Yamamoto GOU, Tsuji M, Oyamada H (2012). Analysis of p53 and miRNA Expression after Irradiation of Glioblastoma Cell Lines. Anticancer Research.

[44] Mehdizadeh R, Madjid Ansari A, Forouzesh F, Shahriari F, Shariatpanahi SP, Salaritabar A (2023). P53 status, and G2/M cell cycle arrest, are determining factors in cell-death induction mediated by ELF-EMF in glioblastoma. Sci Rep.

