# Supplementary material for: TRIM47-mediated Ubiquitination of p53 Controls Proliferative Progression and Stress Adaptation in Glioblastoma
Source: Int J Biol Sci. 2026 Apr 8;22(8):4383–98. doi: 10.7150/ijbs.131392 (PMC13137981; doi:10.7150/ijbs.131392)
Supplement: Supplementary file 1 — Supplementary figures. [file ijbsv22p4383s1.pdf]

Supplementary Materials for

TRIM47-mediated Ubiquitination of p53 Controls Proliferative Progression and Stress Adaptation in Glioblastoma

Dakun Pei *et al.*

*Corresponding author. Email: hongjuan.cui@gmail.com, liangping868@sina.com.

Table.S1 Sequence of the TRIM47 shRNA primers. The shRNA sequences are listed below:

TRIM47#1-F	CCGGGTTTGCCTATATTGTGGATTTCTCGAGAAATCCACAATATAGGCAAACTTTTTG
TRIM47#1-R	AATTCAAAAAGTTTGCCTATATTGTGGATTTCTCGAGAAATCCACAATATAGGCAAAC
TRIM47#3-F	CCGGCCACACACCCAGCCTTCTCATCTCGAGATGAGAAGGCTGGGTGTGTGGTTTTTG
TRIM47#3-R	AATTCAAAAACCACACACCCAGCCTTCTCATCTCGAGATGAGAAGGCTGGGTGTGTGG

Table.S2 Sequence of the PDK1 shRNA primers. The shRNA sequences are listed below:

PDK1#1-F	CCGGGATAAGCGGAAGGGTTTATTTCTCGAGAAATAAACCCTTCCGCTTATCTTTTTG
PDK1#1-R	AATTCAAAAAGATAAGCGGAAGGGTTTATTTCTCGAGAAATAAACCCTTCCGCTTATC
PDK1#2-F	CCGGCAAAGTTCTGAAAGGTGAAATCTCGAGATTTCACCTTTCAGAACTTTGTTTTTG
PDK1#2-R	AATTCAAAAACAAAGTTCTGAAAGGTGAAATCTCGAGATTTCACCTTTCAGAACTTTG

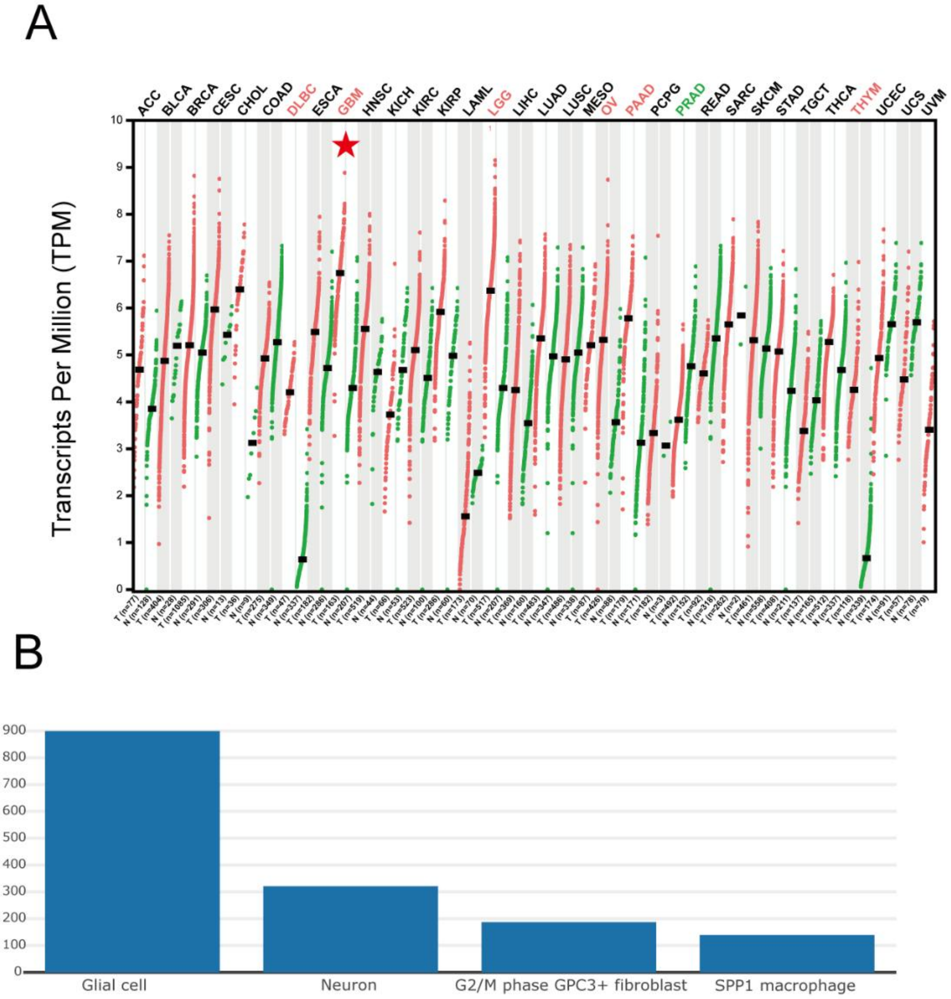

Figure S1. Additional analyses of TRIM47 expression across cancer types and single-cell datasets

(A) Pan-cancer analysis based on The Cancer Genome Atlas (TCGA) dataset showing TRIM47 mRNA expression levels across multiple cancer types, with glioblastoma (GBM) displaying relatively elevated TRIM47 expression compared with most other tumor entities.

(B) Single-cell RNA sequencing analysis from the DISCO database illustrating the distribution of TRIM47 expression across different cell populations within glioma samples, supporting preferential expression in glial-lineage cells.

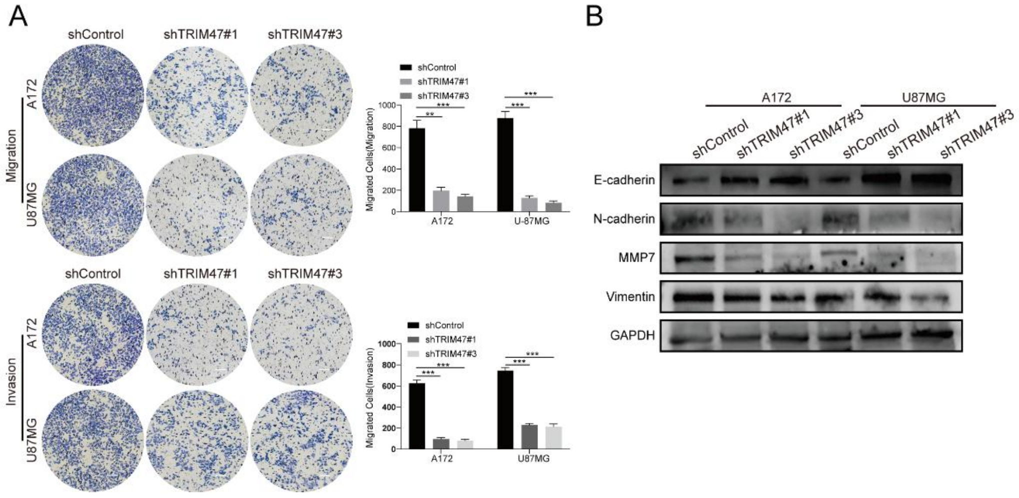

Figure S2. TRIM47 depletion suppresses migration- and invasion-associated phenotypes in GBM cells

(A) Representative images of Transwell migration and invasion assays performed in A172 and U87MG cells following TRIM47 knockdown. Cells transduced with control or TRIM47-targeting shRNAs were seeded into Transwell chambers, and migrated or invaded cells were fixed and stained after the indicated incubation period.

(B) Western blot analysis of migration- and invasion-related proteins in control and TRIM47-depleted GBM cells, supporting reduced motility upon TRIM47 knockdown.

Scale bar = 100 μm.

Data are presented as mean ± SD from at least three independent experiments. Statistical significance was determined using Student's t-test. $P < 0.05$ was considered statistically significant.

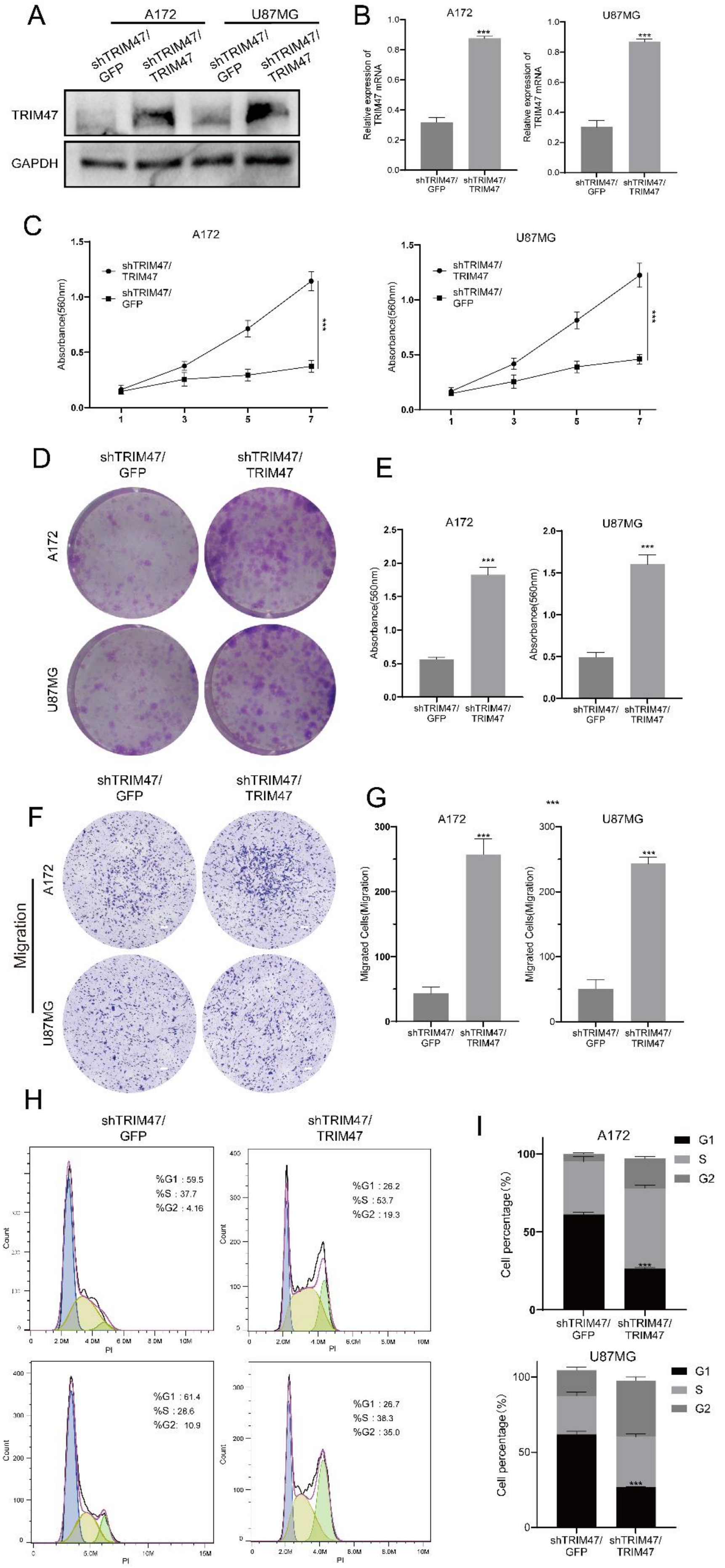
A
A172
U87MG
shTRIM47/ GFP
shTRIM47/ TRIM47
TRIM47
GAPDH
B
Relative expression of TRIM47 mRNA
C
Absorbance(560nm)
D
E
F
Migration
G
Migrated Cells(Migration)
H
%G1 : 59.5
%S : 37.7
%G2 : 4.16
%G1 : 26.2
%S : 53.7
%G2 : 19.3
%G1 : 61.4
%S : 28.6
%G2: 10.9
%G1 : 26.7
%S : 38.3
%G2 : 35.0
Count
PI
I
Cell percentage (%)
G1
S
G2

Figure S3. Restoration of TRIM47 expression rescues proliferative, migratory, and cell cycle phenotypes in GBM cells.

(A–B) Western blot and qRT-PCR analyses of TRIM47 protein and mRNA expression following TRIM47 re-expression.

(C) CCK-8 proliferation assays showing partial restoration of proliferation upon TRIM47 re-expression.

(D–E) Representative crystal violet staining images (D) and quantification (E) showing enhanced colony formation ability after TRIM47 restoration.

(F–G) Transwell migration assays showing recovered migratory potential following TRIM47 re-expression. Scale bar = 100 μm.

(H–I) Flow cytometry analysis of cell cycle distribution demonstrating the rescue of cell cycle arrest after TRIM47 re-expression.

All data are expressed as mean ± SD from at least three biological replicates ($n \geqslant 3$). Statistical significance was determined by Student's t-test ($p < 0.05$).

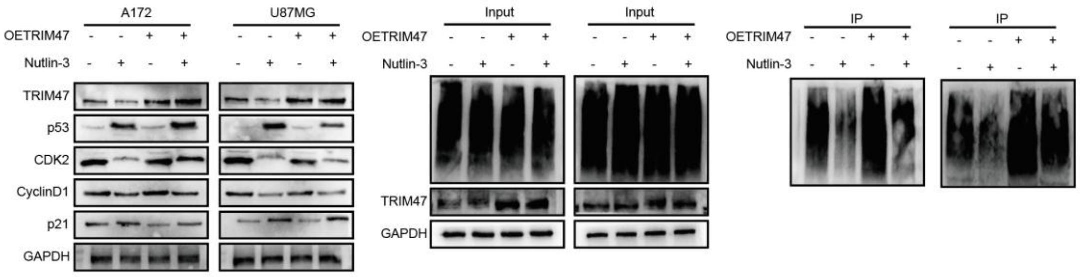

FigS4. Protein levels of TRIM47, p53, p21 and cell-cycle regulators, and p53 ubiquitination status, under TRIM47 overexpression in the presence or absence of Nutlin-3.

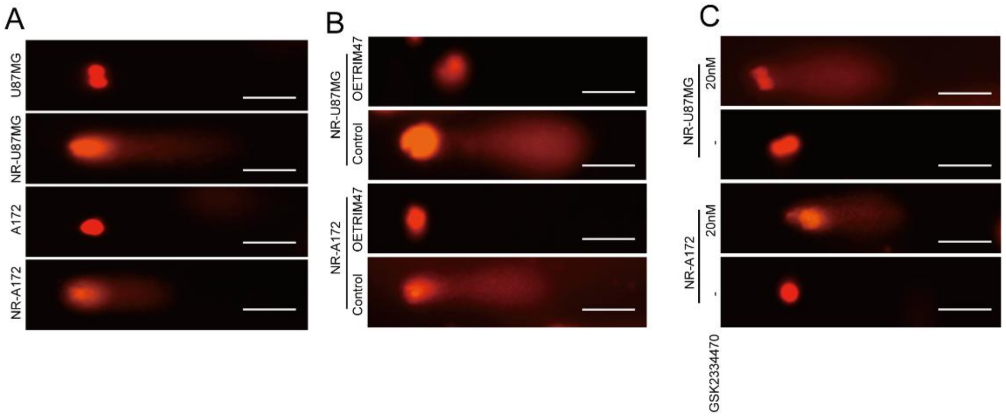

Figure S5. Effects of TRIM47 and PDK1 on TMZ-induced DNA damage in GBM cells

(A) Comet assay demonstrating increased DNA damage in GBM cells under high-dose TMZ treatment. Representative images and quantitative analysis of comet tail moments are shown. Scale bar = 100 μm.

(B) Comet assay showing that ectopic overexpression of TRIM47 attenuates DNA damage in NR-A172 and NR-U87MG cells. Scale bar = 100 μm.

(C) Comet assay showing enhanced DNA damage in NR-A172 and NR-U87MG cells following pharmacological inhibition of PDK1. Scale bar = 100 μm.

Data are presented as mean ± SD from at least three independent experiments. Statistical significance was determined using Student's t-test. $P < 0.05$ was considered statistically significant.
